# An Atlas of Clinical Microscopy

**Published:** 1886-12

**Authors:** Lester Curtis


An Atlas of Clinical Microscopy. *By* Alexander Peyer, M.D., *translated and edited by* Alfred C. Girard, M.D. *New York:* D. Appleton & Company. *Chicago:* Jansen, McClurg & Company.

The book has ninety plates, some of the plates having several different sets of figures. The subjects comprise blood, milk, urine, sputum. fæces, vomit, "contents of abdominal tumors," by which he means one plate devoted to an ovarian cyst, " secretion of female sexual organs," and "various micro-organisms provoking disease."

The plates are carefully, sometimes laboriously, drawn, but with a stiffness of hand which shows that the author is not an artist. In many instances, also, they must have been finished away from the microscope, as shown in the air-bubbles in plate five, which no one would recognize. The text is brief, as such a text should be, but crude and often untrustworthy. For example, one familiar with the subject would not at this day have said, "the relative quantity of the leucocytes to the red corpuscles is 1 to 300" (page 2). Again, the statement that "this form of urethritis [from masturbation] never leads to strictures" (page 102) is not true. See Gross: "Disorders of the Male Sexual Organs."

On the whole, the author has attempted something beyond his strength, but appears to have done his work as well as he could.

The translation is readable, but as a specimen of English is neither elegant nor correct.

The publishers have done their part in good style.

Notwithstanding the faults and deficiencies of the work, it supplies a want, and is worthy of a place in the physician's library. LESTER CURTIS.

